# A fast GNU method to draw accurate scientific illustrations for taxonomy

**DOI:** 10.3897/zookeys.515.9459

**Published:** 2015-07-30

**Authors:** Giuseppe Montesanto

**Affiliations:** 1 University of Pisa, Department of Biology, via Luca Ghini 13, 56126 Pisa, Italy

**Keywords:** Methodology, scientific illustrations, digital drawings, GIMP, Oniscidea

## Abstract

Nowadays only digital figures are accepted by the most important journals of taxonomy. These may be produced by scanning conventional drawings, made with high precision technical ink-pens, which normally use capillary cartridge and various line widths. Digital drawing techniques that use vector graphics, have already been described in literature to support scientists in drawing figures and plates for scientific illustrations; these techniques use many different software and hardware devices. The present work gives step-by-step instructions on how to make accurate line drawings with a new procedure that uses bitmap graphics with the GNU Image Manipulation Program (GIMP). This method is noteworthy: it is very accurate, producing detailed lines at the highest resolution; the raster lines appear as realistic ink-made drawings; it is faster than the traditional way of making illustrations; everyone can use this simple technique; this method is completely free as it does not use expensive and licensed software and it can be used with different operating systems. The method has been developed drawing figures of terrestrial isopods and some examples are here given.

## Introduction

Aiming to express a concept or convey a message, the use of a picture is certainly a clearer and understandable way compared to a text-only description. Images help reading a text and explain immediately what that text would represent. In biosystematics, descriptions of new plant and animal taxa are always combined with figures and plates in order to illustrate the anatomical parts and body details. Such figures are of great value for species identifications. Line drawings are normally used for many species descriptions. They can be produced by inexpensive means (ink and paper), as stated in the Council of Biology Editors’ guide, *Illustrating Science*: “A good pen and ink drawing is pleasing, informative and reproduces well, even on poor grades of paper”. The drawings should be organized to highlight the characters and their details. However, each figure should not be cluttered with too much detail, and there should be a pleasing balance between lines and white space (Winston 1999). When line drawing a body part, one usually makes it larger than requested by the size of the journal page in order to highlight details. The reduced final size will look sharper and small imperfections will usually be minimized (Zweifel 1961). Further notes on the preparation of illustrations for taxonomic papers are discussed in Mayr et al. (1953).

Taxonomists need images of good quality in describing taxa. As a rule, drawings are better detailed than stereo or light microscopes photographs since some details, which are often barely visible in a photograph, may be highlighted. When using photographs, focusing a complete detail requires many photos at a high level of magnification. Then, by using an appropriate software, it is afterwards possible to combine all the images, although the resulting quality is considerably low and require further processing steps.

Nowadays, only digital figures (drawings or photographs) are accepted by the most important taxonomic journals. The traditional way to generate high quality figures and plates for taxonomic papers, is to make pencil drawings first and then ink with high precision technical pens, which normally use capillary cartridge and different line widths. The single figures are then settled on a sheet of paper and each plate is scanned to a digital format. This method is sufficiently quick but may produce imperfect lines or gaps.

Digital drawing techniques that use vector graphics have already been described in literature to support scientists in drawing figures and plates for scientific illustrations (Coleman 2003, 2009); these techniques use a range of software packages and various hardware devices, such as digitiser boards or digital graphic pens.

The GNU Project (GNU is a recursive acronym meaning “GNU’s not Unix”) is a mass collaboration project of free software. Software that have been developed under the GNU Project guarantee these freedom-rights legally (via its license), and are therefore free software. GIMP is an acronym for GNU Image Manipulation Program. The GIMP is suitable for a variety of image manipulation tasks, including photo retouching, image construction and composition. GIMP can be used as a simple paint program, or as an expert quality photo retouching program, or again as an online batch processing system, a mass production image render, an image format converter, etc. (GIMP documentation is available at: http://www.gimp.org/). GIMP is freely available from many sources for many operating systems. Most GNU/Linux distributions include the GIMP as a standard application. The GIMP software application is covered by the General Public License (GPL).

The purpose of this methodological work is to describe a new digital drawing method using GIMP software that helps the taxonomist in making high quality line drawings. This method was developed on drawings of terrestrial isopods for taxonomic papers. Some examples are here given.

## Method

First of all GIMP has to be installed on a computer. Notes about installing GIMP on your computer are reported on the official GIMP website at the following URL: http://www.gimp.org/docs/. GIMP version 2.8.14 on a Mac computer (Mac Mini, late 2013, with Mac OS 10.10.1) has been used to illustrate the procedures of this paper.

The pencil drawings that need to be digital traced must be saved in a digital file or directly digitized in a Portable Document Format (a common PDF file).

### Preparing the workspace

In order to prepare the window visualization at first access after the GIMP installation, all the panels that are shown should be closed. Only the “Toolbox” and the Dockable Dialogs “Layers” and “Tool Options” will be used and kept on screen:

Windows > Toolbox

Windows > Dockable Dialogs > Layers

Windows-> Dockable Dialogs > Tool Options.

**Figure 1.**
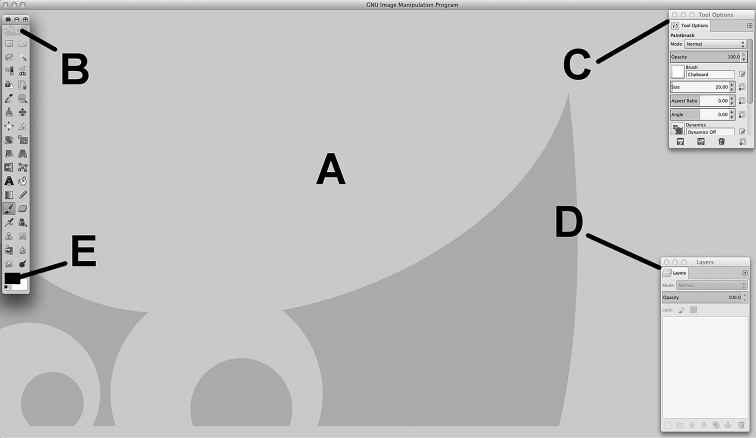
GNU Image Manipulation Program (GIMP, ver. 2.8.14) on a Mac OS. **A** Image window **B** Toolbox window **C** Tool Options window **D** Layers window **E** Foreground (black) and Background colours (white).

Now the workspace should be arranged as reported in Fig. 1, with Image window (Fig. 1A) and three floating windows: Toolbox (Fig. 1B), Tool Options (Fig. 1C) and Layers (Fig. 1D). In the Toolbox select black and white for the Foreground and Background colours (Fig. 1E).

**Figure 2.**
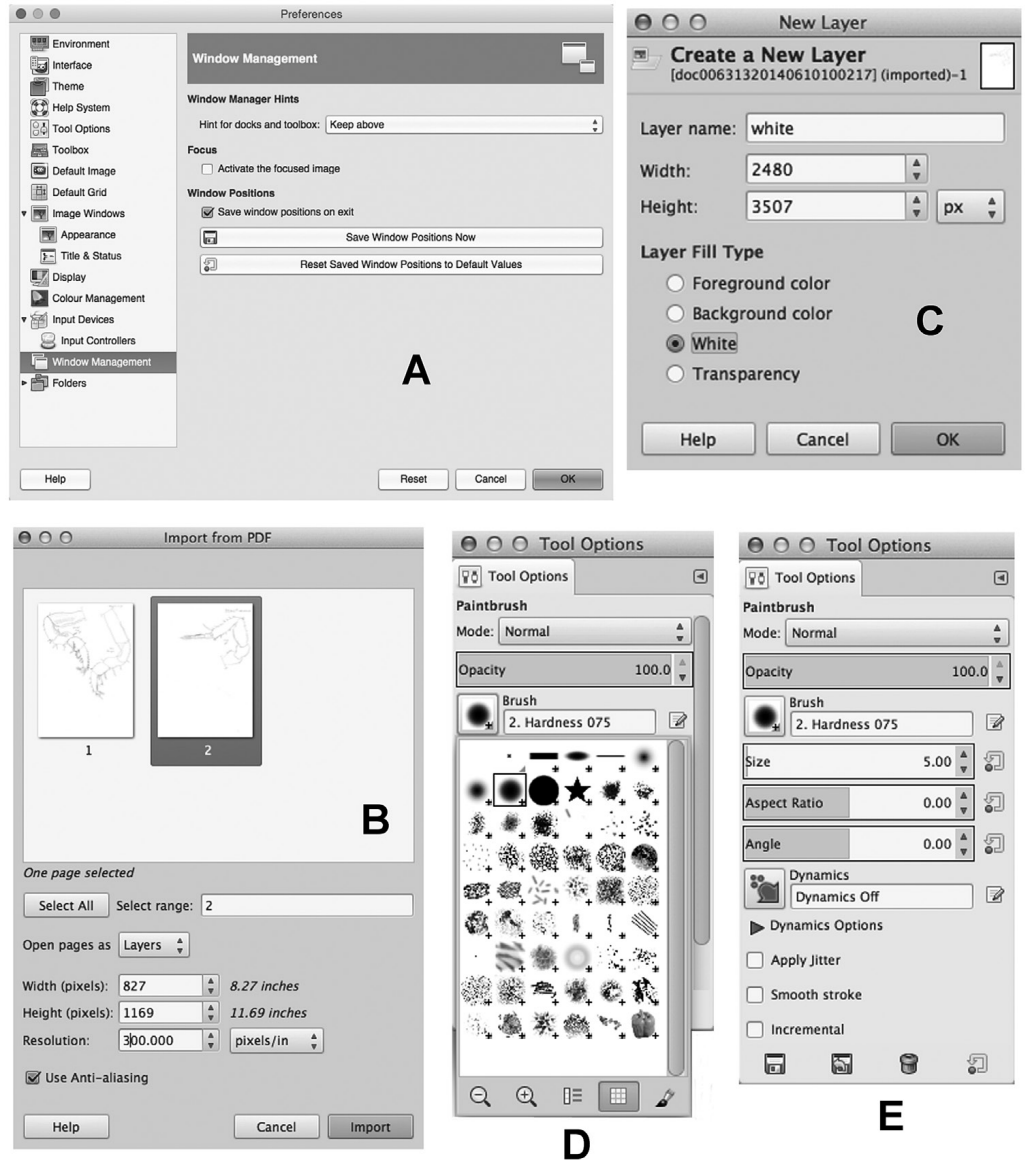
GNU Image Manipulation Program. **A** Preferences window, with the “Window Management” settings **B** “Import from PDF” window appears after a PDF file has been opened with the File > Open command of menu **C** New Layer window with the Layer Fill Type option set on “White” **D** Tool Options window of Brush Tool with the brush No. 2. Hardness 075, used in the present drawing method **E** Tool Options window (Brush Tool) with the brush size set on 5 pixels (5.00).

Using different versions of GIMP or different operative systems the toolbox and the two Dockable Dialogs are sometimes covered by the Image window and not visible as front windows. In GIMP for Windows OS you can change this setting from: Edit > Preferences > Window Management. In Mac OS from: GIMP > Preferences > Window Management (Fig. 2A). To have the toolbox and the other windows always on top of the screen change the Hint for the toolbox from ‘Normal Window’ to ‘Utility Window’. You will be asked to restart GIMP in order to make changes effective.

### Step-by-step instructions

The user can follow the instructions of the present method according to two different levels of difficulty: beginner and advanced. Beginners are suggested to start testing the former method and then move to the advanced level once they have gained some experience.

### Beginner level

1. Open the file that contains the pencil drawings: File > Open
  (with PDF files, set the resolution at 300 pixel/in) (Fig. 2B).
2. Create a new layer with a white background:
  Layer > New Layer
  (Layer Set “White” as Layer Fill Type) (Fig. 2C).
  Set the opacity of this new level to 60–80 in the Layers window.
3. Create another new layer for the first trace: Layer > New Layer
  (Set “Transparency” as Layer Fill Type).
4. Choose the brush left clicking on “Paintbrush Tool”.
  On the “Tool Options” window, click on the square icon labelled “Brush” (Fig. 2D).
  Choose the brush No. 2. “Hardness 075”.
  Set the “Size” to 3-5 points (Fig. 2E). Adjust the zoom level to 400–800%
5. Start drawing (see the “How to draw” section).
6. Once finished, set again the opacity of the “White” layer to 100, and the opacity of this first trace layer to 20–40, in order to better see the second trace.
7. Create another new layer for the second trace: Layer > New Layer
  (Set “Transparency” as Layer Fill Type).
8. Start drawing in this new layer, following only the lines in the first trace. Remember to set the brush size to 5–7 points and to adjust the zoom level to 800–1100%.

### Advanced level

1. Open the file that contains the pencil drawings: File > Open
  (with PDF files, set the resolution at 300 pixel/in)
2. Create a new layer for the trace: Layer > New Layer
  (Set “Transparency” as Layer Fill Type).
3. Choose the brush: click on “Paintbrush Tool”. On the “Tool Options” window click on the square labelled “Brush”. Choose the brush “2. Hardness 075”. Set the “Size” to 5–6 points.
4. Start drawing (see the “How to draw” section), following the lines of pencil drawings.

### How to draw (with a mouse)

**Figure 3.**
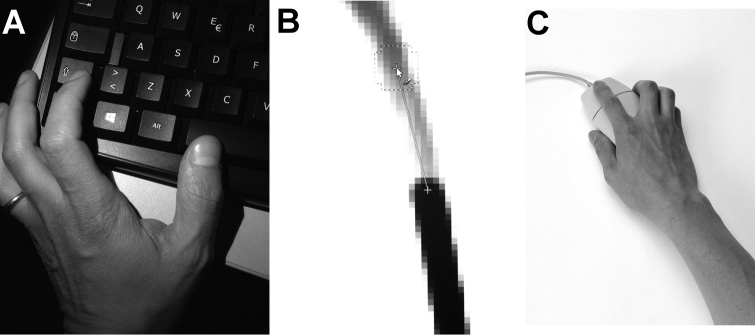
How to draw lines with GNU Image Manipulation Program. **A** Left hand position: with Shift key pressed to draw little segments, space bar to move the screen visual along the drawing **B** Portion of the Image window (at 800% zoom level) showing the drawing guide-line **C** Right hand position with a common mouse.

- Set the zoom level to 400–800% for the first trace, 800–1100% for the second (or at advanced level);
- choose an appropriate starting point (e.g. a corner or an intersection);
- choose the brush tool with the right size (see above) and do a first click with the left button (Fig. 3C) at the starting point you have chosen;
- start to trace out the pencil line by pressing the shift key on the keyboard (Fig. 3A), then move the mouse pointer along the pencil line: a guide line will appear to help you to trace a segment (Fig. 3B);
- stop the pointer where you consider worthwhile; left click once again to trace a segment;
- press the space bar of the keyboard (Fig. 3A) to move along the drawing, only moving the mouse, without clicking any other button;
- change the brush size whenever you need to draw smaller details;
- find a suitable balance between several closer points or less numerous but more distant points (i.e. the suitable balance between the time you have and the quality of your drawings).

Shorter segments will appear as a continuous line and the quality will be even better in the printed version since the figure size is smaller than the digital image.

### Drawing dashed lines

**Figure 4.**
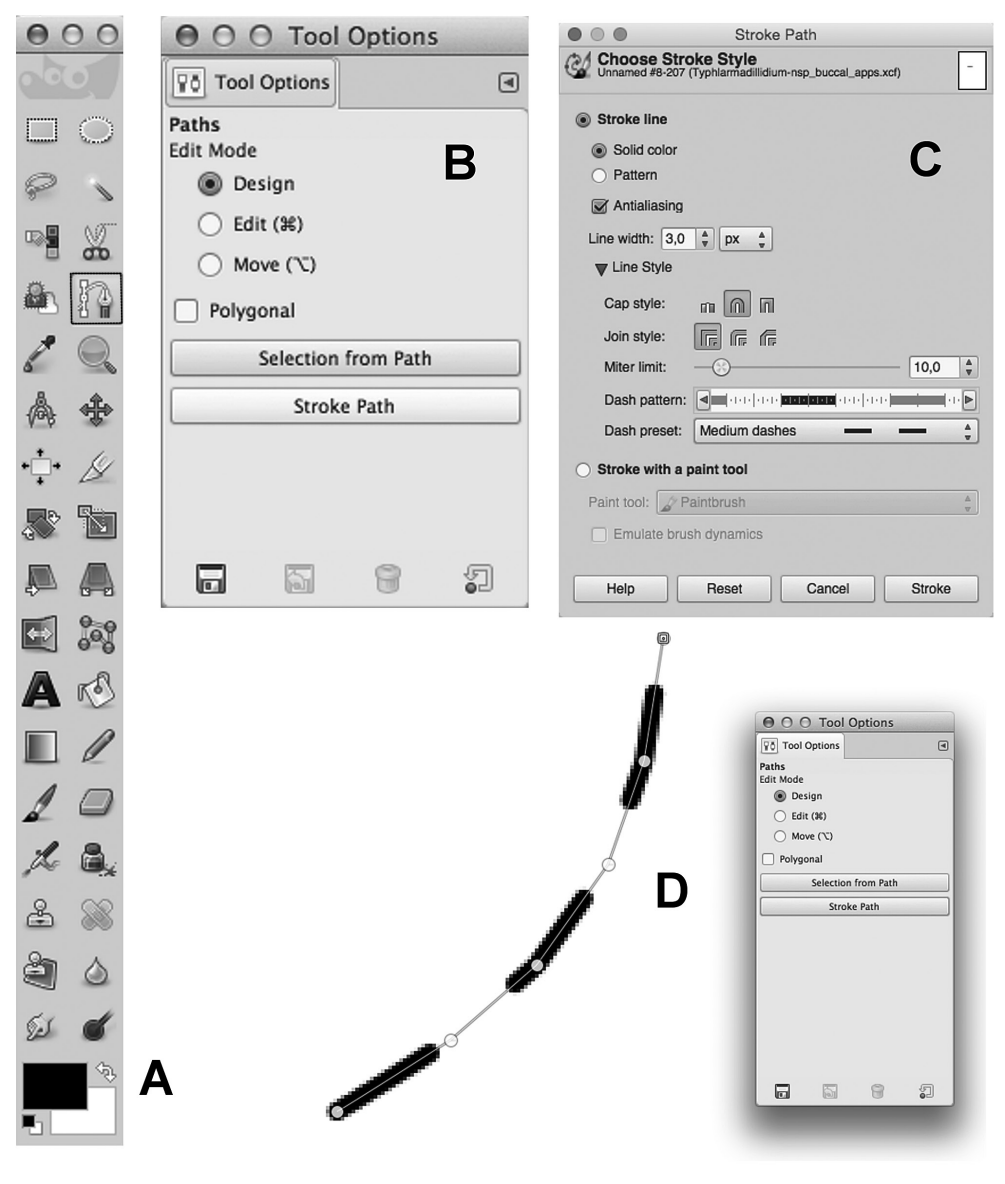
GNU Image Manipulation Program. **A** Toolbox window: Path Tool is marked with a black square **B** Tool Options window of Path Tool **C** Stroke Path windows with the settings for a “Medium dashed” line (3 px) **D** Portion of the Image window showing the result (at 400% zoom level) of the “Stroke Path” button.

- Choose the “Path tool” in the toolbox (Fig. 4A);
- left click several times following the pencil line;
- once finished, click “Stroke path” in the “Tool options” dialog window (Fig. 4B);
- in the “Stroke path” window (Fig. 4C), choose the line width (e.g. 4 or 5 pixels) and open the “Line Style” dialog;
- choose “round” (the second icon) for “Cap style” in the “Line style” option, then “Medium dashed” in the “Dash preset” option (Fig. 4C);
- click on the “Stroke” button and then again on the “Paintbrush tool” to see the result (Fig. 4D).

### Sorting out plates

**Figure 5.**
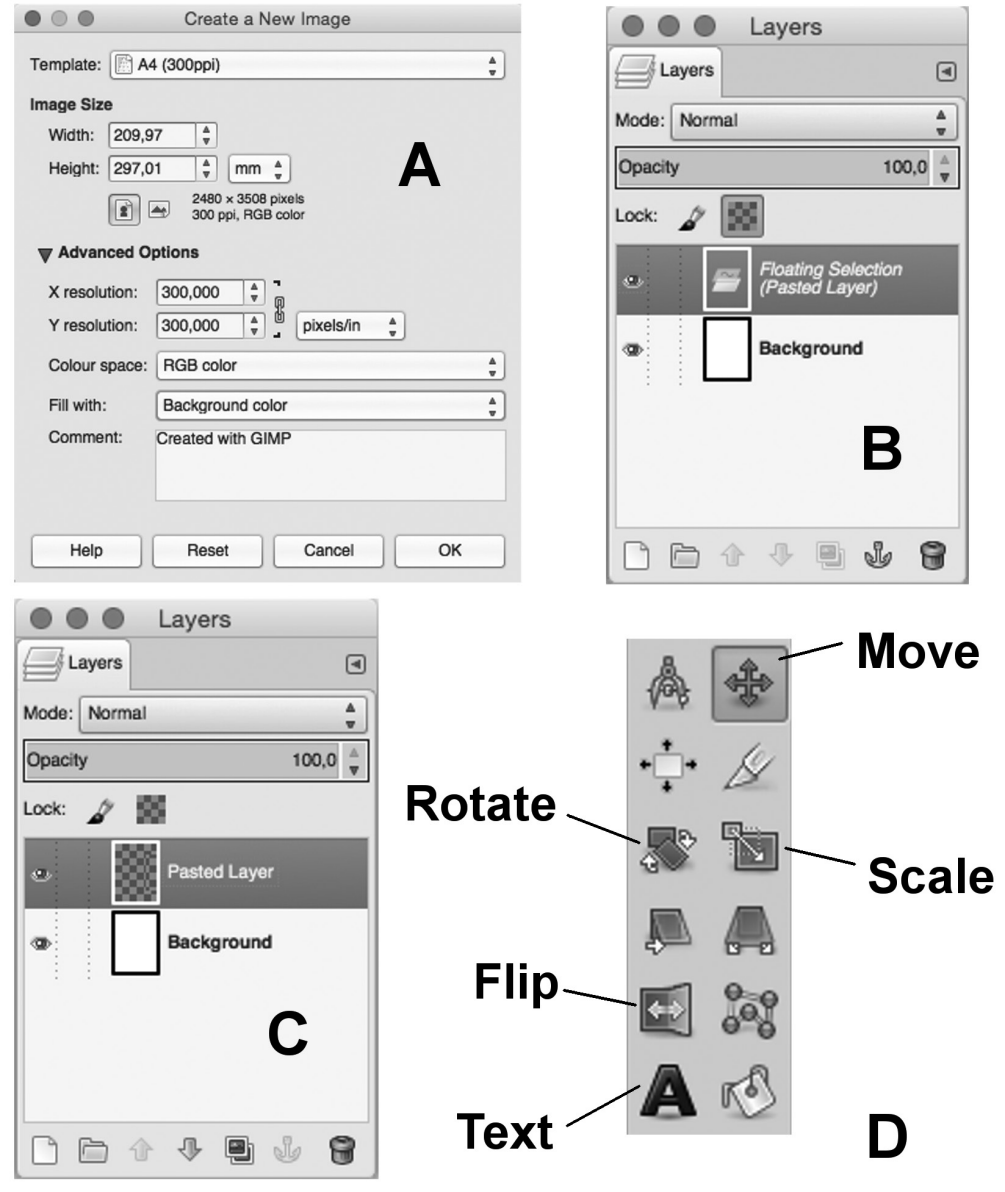
GNU Image Manipulation Program. **A** “Create a New Image” window with settings for a blank page in international A4 format **B** Layers window after a trace was pasted into a new image, the “Floating Selection” layer is showed **C** “Pasted Layer” on the same window after the “Floating Selection” was transformed in a new layer **D** Portion of the Toolbox window showing the tool icons (explanation in the text).

First of all open a new file with a blank image. Choose File > New and select A4 (300 ppi) in the Template menu, then click “OK” (Fig. 5A). The size of this new image will be 2480 × 3508 pixels at 300 points per inches. It is possible to change the resolution, e.g. at 600 ppi, opening the Advanced Options (Fig. 5A)

In order to move the trace that has been already drawn into the new plate: open the. xcf file and select the layer that contains that trace. Use the ‘Free Select Tool’ to carve out the part of the drawing; left click several times following the trace outline. Then copy the selection (Edit > Copy) and paste it (Edit > Paste) into the new blank image. A new layer, named ‘Floating Selection’ will appear in the Layers Dialog (Fig. 5B); right click on this new layer and choose ‘To a new layer’ to create a new layer (named “Pasted Layer”) with the clipping (Fig. 5C). This is particularly useful in order to rearrange the plate later. It is also possible to move the entire layer (with a simple drag and drop of the layer into the new image) but this step will increase the file size. The new layer can be renamed by double-clicking on its name in the “Layers” window.

Next step is to move, scale, flip or rotate the drawings to set them in the right position. In the toolbox it is possible to find the right instruments (Fig. 5D).

*Move.* Use the “Move Tool” in the Toolbox. In the “Layers” window select the layer with the trace. In the Tool Option Window, choose “Layer” (the first icon) in the “Move” option and “Move the active layer” in the “Tool Toggle” option. Then, it is possible to move the trace in the right position by left clicking on the trace and dragging the layer.

*Scale.* Use the “Scale Tool” in the Toolbox. In the Tool Option Window, choose “Layer” in the “Transform” option and leave the other options with the default values. Left click on the trace and a grid and a “Scale” window will appear helping the user to adjust the image at the right size. It is possible to use percentages by changing the “pixels” option.

*Flip.* Similarly, use the “Flip Tool”. In the Tool Option Window, choose “Layer” in the “Affect” option and then choose “Horizontal” or “Vertical” in the “Flip type” option. Left click on the trace and the drawing will be mirrored upside down or left/right.

*Rotate.* It is also possible to rotate a drawing with the “Rotate Tool”. As before, left click on the trace and a grid and a “Scale” window will appear helping the user to adjust the image at the right angle. Then left click on the “Rotate” button to set the new arrangement.

Once completed, it is necessary to repeat the steps above to insert all the drawings into the new plate.

*Lettering.* Easily, it is possible to add text (e.g. numbers and letters) on the new plate using the “Text Tool”. A common option in the “Tool Options” window is: “Arial” in the Font box and 80–100 in the Size box. A new layer will appear with the text. It can be easily moved with the “Move Tool” just as explained before.

It is also possible to crop the image in order to eliminate the blank space around the traces.

This can be easily done with the “Crop Tool” in the Toolbox. Left-click and drag the mouse pointer to crop the part of the plate.

### Saving the files

**Figure 6.**
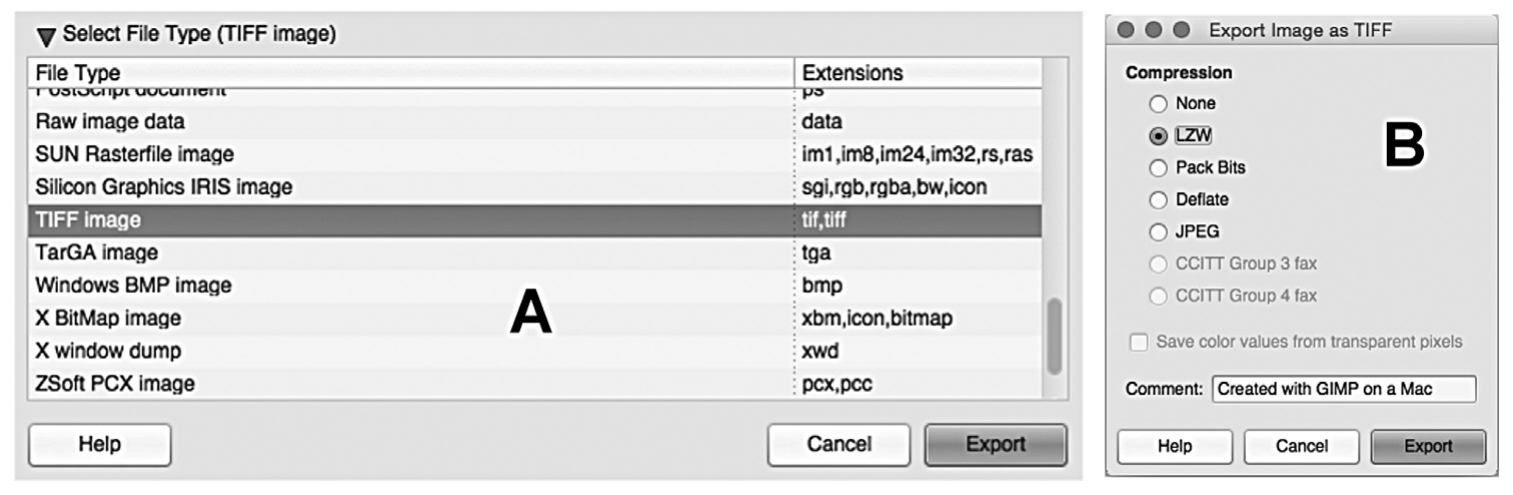
GNU Image Manipulation Program. **A** Portion of the “Export Image” window, showing the “Select File Type” menu with the settings for a TIFF image **B** “Export Image as TIFF” window, with the settings for LZW compression.

Once completed it is important to save the work in different files. Precisely, the user should be save the draft files in the GIMP proprietary format XCF (eXperimental Computing Facility) in order to leave the possibility to work again on the different layers. In the “File” menu, choose “Save” (or “Save As”); then the user will be asked to add a file name and select a destination folder. Otherwise, it is possible to save the plates as one-layer files in a high resolution format, such as TIFF files, normally used for publication in a scientific journal. This step should be done with the “Export” (or “Export As”) option of the “File” menu. A new dialog window will appear on the screen: in this case choose “TIFF image” in the “Select File Type” option (Fig. 6A). Then, it is possible to set the level of compression (that is the quality of images). Generally a LZW compression offer the right balance between quality of the printed image and an appropriate file size. This option is specifically requested by several journals (Fig. 6B).

## Results

The method here explained, produce three different kind of files: the original digitized pencil drawings (normally PDF or TIFF files), the. xcf files of the traces, the. xcf files of the plates. These last ones are used to export the plates in ‘Tagged Image File Format’ (TIF) that may be directly uploaded to the journal submission website. All those files should be kept because they could be useful in the future for many purposes.

The final result is to have perfect lines without any signs of tremble. The black lines appear as a sharp continuous line in the final version, because the size of the printed image is not as big as the digital image. Generally, the use of digital drawing techniques allows undoing the last action and trying again after an error, until the result is satisfying.

**Figure 7.**
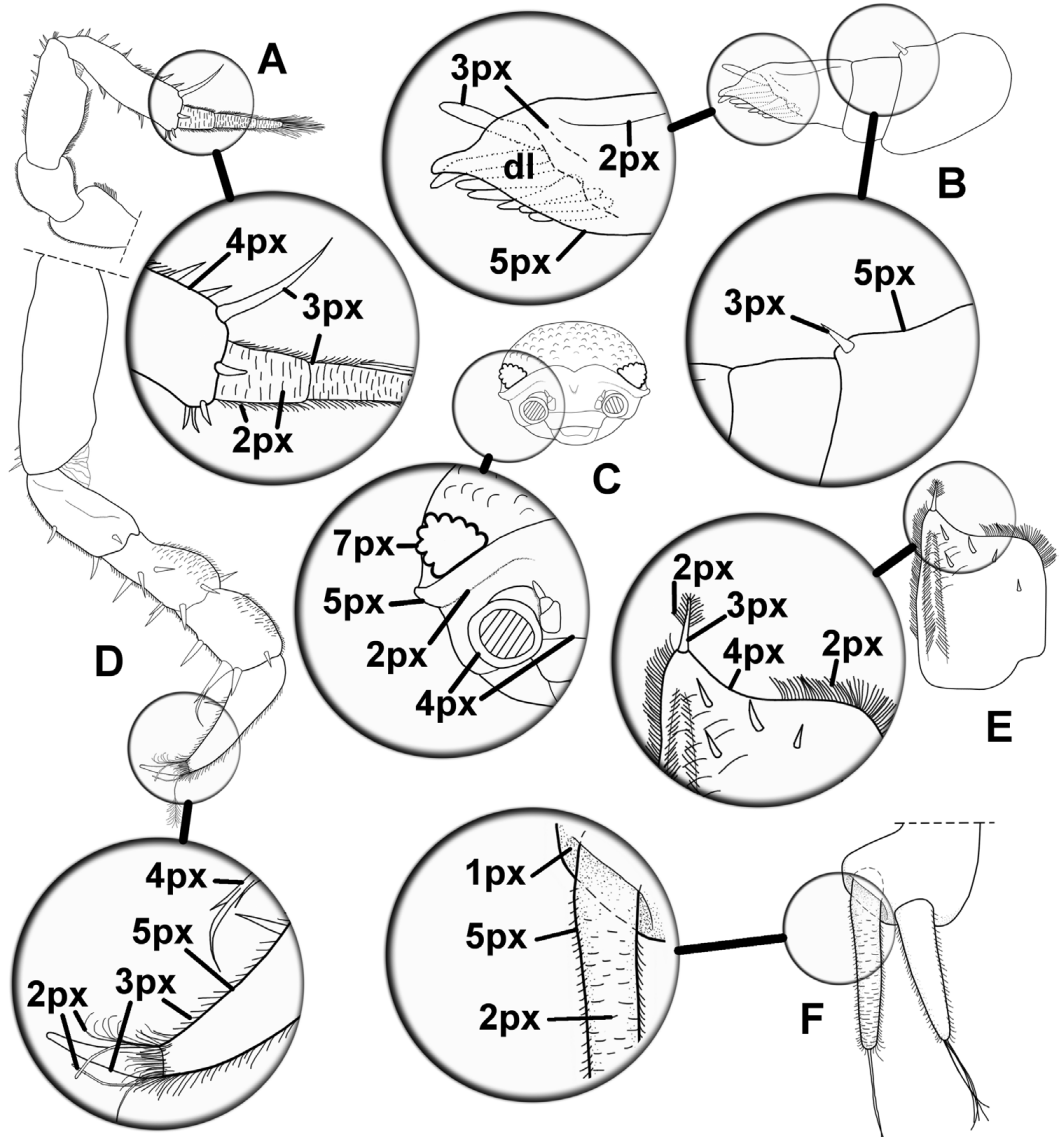
Examples of line drawings of terrestrial isopods anatomical parts. Magnifications in the black circles indicate the brush size (see also the “How to draw” section). **A** Antenna **B** Antennula **C** Cephalon (front view) **D** Pereopod 1 **E** Exopod of pleopod 4 **F** Uropod; dl, dotted lines (Commands: Paths Tool > Stroke Path in the Tool Options > Line style, Dash preset: Dense dots).

Some illustrations made with the method of the present paper, were already published in other articles about terrestrial isopods: figure 1 in Medini-Bouaziz et al. (2006); figure 2 in Montesanto et al. (2007); figures 2, 3 in Montesanto et al. (2008); figures 1–5 in Montesanto et al. (2011); figures 1, 2 in Messina et al. (2011); figure 1 in Messina et al. (2012); figures 6, 7, and 9 in Montesanto et al. (2012); figure 1 in Lupetti et al. (2013); figures 2, 3 in Montesanto et al. (2013); figures 1–6 in Messina et al. (2014). Some indications about drawing terrestrial isopods for taxomomic papers are reported in Figure 7, whereas magnifications show the differences of the brush size for different anatomical parts.

Finally, due to the practice made with the method here introduced, some other suggestion are proposed to the reader: draw the possible dotted shadows on a new layer above the other ones; use maximum zoom level for details, even more than 1100%; delete errors with the Eraser Tool (in the toolbox) or by clicking “Undo” in the Edit menu; always look at the general shape of the curve (reducing zoom level); avoid excessively long segments in order to point out no edges; try different size of brushes for setae, spines and other little details (see Fig. 7).

## Discussion

In a work of biological taxonomy, the part that requires more time, is the preparation of illustrations. There are always many species that need to be described so some taxonomists usually describe more species, not so detailed, others describe fewer species, but very detailed (Coleman 2006). Our predecessors and contemporary isopodologists published exemplary literature (e.g. Vandel 1960, 1962; Caruso and Lombardo 1978, 1982; Schmalfuss 1996; Taiti and Ferrara 1996; Schmidt 2002, 2003, 2007; Taiti and Argano 2009; Taiti and Gruber 2010); every taxonomist would aim to publish such high quality works that would be still useful in the future, but the limited time is always a problem. Software packages like DELTA (Dallwitz et al. 1993; Dallwitz et al. 1998), can automatically generate dichotomous keys and also taxonomical diagnoses and descriptions, that accelerate the description process. Nevertheless, making illustrations for taxonomy papers and also for software like DELTA, is always a long process (Coleman 2006). At first a pencil drawing is made often using a camera lucida; then these drawings are traced again on plates for the publication.

Other similar methods of digital drawings techniques, but with the use of vector graphics, have already been described in literature (Bouck and Thistle 1999, Coleman 2003, Bober and Riehl 2014). Such methods were a significant step forward in speeding up the time consuming part of a taxonomic description, but they nevertheless showed some weak spots. Actually, those techniques require the use of several software and various hardware devices (such as digitiser boards and digital graphic pens), and plus the cost of complicate and very expensive software (e.g. Adobe Illustrator™, Adobe Photoshop™). Nonetheless, simple and free vector graphics software (e.g. Inkscape, https://inkscape.org) may be used in order to obtain good results.

On the other hand, the method here discussed produces good results in terms of image quality and precision, saving time and costs. Specific strengths are listed here: 1. It is simple but accurate, producing detailed lines at the highest resolution. 2. Small structures can be greatly magnified, so that drawing is easier than using common inking on paper. 3. The raster lines appear as realistic ink-made drawings. 4. It is much faster than the traditional way of making illustrations. 5. Everyone can learn to use this simple technique; it can also be used by technical staff or even inexperienced volunteers. 6. This method is completely free as it doesn’t use expensive and licensed software. 7. As reported in the introduction of this article, GIMP is available for different platforms (Windows, Mac OS, or Linux), thus the work files can be moved in different computers maintaining the same file extension (.xcf).

Genuinely, some specialist can find the last points debatable preferring, therefore, the use of vector graphics. For example, only vector graphics allows to scale a drawing supporting the maximum detail; on the other hand when bitmap line drawings are magnified over 200%, they clearly show a typical ‘pixels’ vision. In addition, some other specialist can find a better choice working with a drawing board instead of a simple mouse, and this is also possible with GIMP. These are clearly subjective points. The best suggestion is try as many methods as possible. Then, once practiced, the users will have their better choice.

The basis for the digital drawing method here proposed is a conventional pencil drawing, made with a microscope and a camera lucida. However, it is possible to use the same method starting with a work of stack microphotography, in order to avoid the time-taking drawing process. For this purpose many software are available to combine images of different depth of focus into one photo, such as Auto-Montage (http://www.syncroscopy.com/auto-montage/), CombineZP (http://www.hadleyweb.pwp.blueyonder.co.uk/), Helicon Focus (http://www.heliconsoft.com/), Zerene Stacker (http://www.zerenesystems.com/). Recently, an interesting comparison of the focus stacking software packages has been published by Brecko et al. (2014), as a possible solution for mass digitization of type specimens; an ant of the genus *Meranoplus* and a beetle of the genus *Trachys* were tested. Nonetheless, these photographic methods show some issues (see also Coleman 2006). Actually, a drawing can show important characters that are difficult to see in a photo. Even small details, such as aestetaschs of antennulae, so important for terrestrial isopods taxonomy, can be easily shown, e.g. in Trichoniscidae, fine lines between articles of antennal flagellum, or other characters that are often used for species taxonomy. A detailed illustration may be considered as an interpretation, it is not only a simple description of the morphology. So, it is possible to point out some structures that are covered by others (Coleman 2006). Generally, one other big advantage of such digital inking methods is that technical assistants or other volunteers may be employed in order to save time. The specialist can afterwards correct the illustrations, if indispensable (see also Coleman 2003, 2006).

GIMP offers many other opportunities that have not been described in this paper. In fact, the use of GIMP has been cited in other papers on digital drawing (such as: Sidorchuk and Vorontsov 2014). Here have been reported only the essential informations needed to obtain high-quality figures and plates which are suitable for online and printed publication. Any further request for clarification may be asked to the author.

### Additional material

An explanatory playlist (with 6 videos) has been published at the following URL, in order to facilitate the users with the method here showed:

https://www.youtube.com/playlist?list=PLHuMNpWqA6OxGAgzk6yp07a55KV7i2caJ
